# Asthma in adolescence affects daily life and school attendance – Two cross‐sectional population‐based studies 10 years apart

**DOI:** 10.1002/nop2.77

**Published:** 2017-03-08

**Authors:** Caroline Stridsman, Elisabeth Dahlberg, Karin Zandrén, Linnéa Hedman

**Affiliations:** ^1^ Department of ResearchThe OLIN UnitNorrbotten County CouncilLuleåSweden; ^2^ Division of NursingDepartment of Health SciencesLuleå University of TechnologyLuleåSweden; ^3^ Department of Public Health and Clinical Medicine, Occupational and Environmental MedicineThe OLIN UnitUmeå UniversityUmeåSweden

**Keywords:** absenteeism, adolescents, asthma, environment, epidemiology, nursing, physical education, school nursing

## Abstract

**Aim:**

The aim of this study was to study the impact of asthma on daily life, school absenteeism and physical education. In addition, to describe asthma triggers at school.

**Design:**

Two cross‐sectional population‐based studies ten years apart.

**Method:**

Within the OLIN‐studies, in 2003 (*n *=* *3,327) and in 2013 (*n *=* *2,345) adolescents (14–15 years) answered an expanded ISAAC questionnaire. Of these, 8% and 11%, respectively with current asthma participated in this study.

**Results:**

Between the years 2003–2013, the proportion of adolescents reporting that asthma interfered with daily life had increased, in 2013, girls were significantly more affected than boys. The proportion reporting a worsening of asthma at school had decreased, but it was still over a quarter. The proportion of absenteeism from school and from physical education was at the same level both years. Asthma triggers were described to be poor air quality, poorly cleaned environment, allergens, strong fragrance, rebuilding projects, physical education and stress.

## 1 Introduction

Asthma is a chronic disease and a global health problem that affects people of all ages. Worldwide, approximately 235–300 million people suffer from asthma (Masoli, Fabian, Holt, & Beasley, 2004; World Health Organization (WHO), 2013) and among children, asthma is the most common chronic disease (WHO, 2013). Compared with their healthy peers, schoolchildren with asthma seem to have an impaired health status as assessed by quality of life questionnaires (Merikallio, Mustalahti, Remes, Valovirta, & Kaila, 2005). There are also studies showing that health status among adolescents with asthma is worse among girls than boys (Rydström, Dalheim‐Englund, Holritz‐Rasmussen, Möller, & Sandman, 2005; Sundell, Bergström, Hedlin, Ygge, & Tunsäter, 2011). Girls express a higher degree of feelings such as frustration, being left out, anger, worry, concern, discomfort and fear from asthma attacks. Nevertheless, Sundell et al. (2011) reported that in both sexes, self‐rated health status improved with increasing age.

There is a relationship between high body mass index (BMI) and the presence of wheezing and asthma among schoolchildren (Mai et al., 2003). Conversely, high levels of physical activity seem to be a possible protective factor against asthma development (Eijkemans, Mommers, Draaisma, Thijs, & Prins, 2012). Furthermore, physical activity has a positive effect on academic performance (Fedewa & Ahn, 2011), improves cardiopulmonary fitness (Ram, Robinson, Black, & Picot, 2005) and has a positive effect on health status among girls with asthma (Sundell et al., 2011). There is an ongoing discussion on whether or not the level of physical activity differs between asthmatic children and their healthy peers. Some researchers argue that there is no difference in physical activity level (Nystad, Nafstad, & Harris, 2001) while others claim that asthma interferes with children's ability to participate in vigorous physical activities (Chiang, Huang, & Fu, 2006). However, children with asthma report that running, bicycling, playing football and physical education are activities that are restricted due to their illness (Rydström et al., 2005).

School absenteeism can affect the learning process negatively and children with asthma seem to have a higher school absence rate than their healthy peers (Bonilla et al., 2005; Moonie, Sterling, Figgs, & Castro, 2006; Silverstein et al., 2001). One explanation may be that school absence is related to the school environment and although schools often have adequate ventilation, the school building is a major site of exposure to cat and dog allergens (Perzanowski, Rönmark, Nold, Lundbäck, & Platts‐Mills, 1999). After indirect exposure to cats (through fur) at school, children with asthma had a higher risk for asthma exacerbations (Almqvist et al., 2001). It has also been shown that the incidence of asthma increased among children attending schools with more settled dust (Smedje & Norbäck, 2001). Other factors in the school environment related to asthma are larger schools, open shelves, low room temperature and high relative air humidity (Smedje, Norbäck, & Edling, 1997).

The Global Initiative for Asthma (GINA) program (2015) and the WHO (2013) are supporting actions to identify and address environmental factors that can reduce the burden of asthma. Adolescents’ daily lives are strongly connected to time spent at school and therefore, it is important to identify asthma triggers in the school environment, as well as the impact asthma has on adolescents’ physical education at school.

## 2 Aims

The aim of this study was to investigate daily life at school among adolescent boys and girls with asthma in 2003–2013 by studying the impact of asthma on daily life, school absenteeism and physical education. We hypothesize that among 14–15 years old adolescents, the disease and its symptoms interferes to a lesser extent on daily life the latter year. A further aim was to describe asthma triggers at school environments in 2003.

### 2.1 Research questions

- Between the years 2003–2013, were there any changes of the impact of asthma on daily life, worsening of asthma in school, school absenteeism and physical education?
- Where there any sex differences?
- Which asthma triggers in the school environment were described by the adolescents?

## 3 Methods

### 3.1 Study design

Two cross‐sectional population‐based studies 10 years apart: 2003–2013.

### 3.2 Data collection procedure

The adolescents under examination were recruited from the Obstructive Lung Disease in Northern Sweden (OLIN) pediatric studies; cohort I and cohort II. The study design of these longitudinal cohort studies and an overview of the results have been described previously (Rönmark et al., 2008). In short, in 1996 and in 2006, respectively, a parental questionnaire mainly about respiratory symptoms, asthma, allergic diseases and their potential risk factors, was distributed to all children in the first and second grades of three municipalities in Northern Sweden and 3,430 (97% in 1996) and 2,585 (96% in 2006) participated (Rönmark, Bjerg, Perzanowski, Platts‐Mills, & Lundbäck, 2009). Both cohorts have been followed up on several occasions, including a questionnaire survey at the age of 14–15 years.

### 3.3 Participants

In the current paper, cross‐sectional data from the surveys taken from adolescents at age of 14–15 is presented (2003: invited *n *=* *3,511, participated *n *=* *3,327 and 2013: invited *n *=* *2,657, participated *n *=* *2,345). The study population consisted of the adolescents who reported “current asthma” (2003 *n *=* *270 and 2013 *n *=* *266), defined as physician‐diagnosed asthma with either current wheezing or asthma medications taken during the last 12 months.

### 3.4 Questionnaire

The questionnaire included the International Study of Asthma and Allergies in Childhood (ISAAC) core questionnaire (Asher et al., 1995). The questionnaire was expanded with additional questions about symptoms, use of medications and physician diagnoses as well as possible risk factors including school environment and physical activity (Rönmark et al., 2009). In 2003, the adolescents also answered an open question: ‘If your asthma gets worse, what in the school setting do you think is causing the deterioration?’

### 3.5 Statistical analyses and analysis of the open question

The Statistical Package for the PASW Statistics (version 20.0; SPSS Inc., Chicago IL, USA) was used for the statistical analyses. Bivariate comparisons were made using Chi square tests and a *p* <.05 was considered statistically significant for all tests. One of the questions regarding school environment was an open question. To produce quantifiable data from this question, a manifest content analysis was conducted (Catanzaro, 1988). The free‐texts were read through several times, categorized and counted together when they had similarities. The categorizing was conducted until there was nothing left to merge and the result describes the adolescents’ experiences of what in the school setting causes deterioration.

### 3.6 Ethics

Informed written consent was given by parents or guardian at the beginning of the studies and by the adolescents when they completed the questionnaire. The studies were approved by the Regional Ethical Review Board at Umeå University, Sweden (99‐408 and 2012‐469‐31M).

## 4 Results

### 4.1 Daily life at school

**Figure 1:**
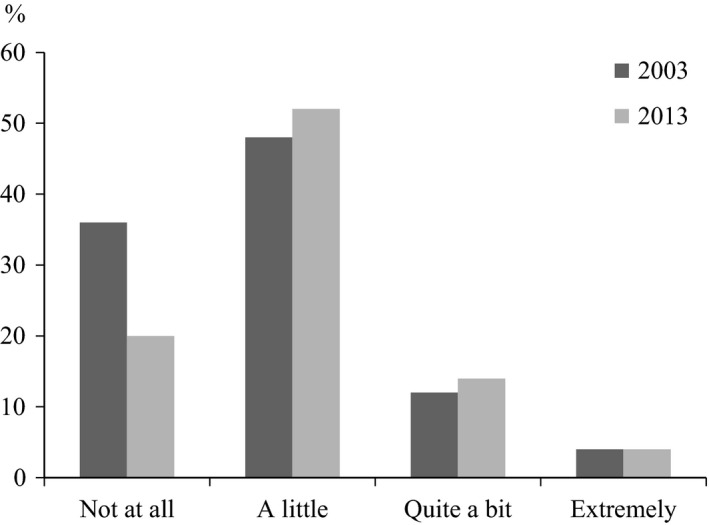
Proportion (%) of how much respiratory symptoms/asthma interferes with daily life reported by adolescents with asthma (14–15 years old) in 2003 and 2013. Test for trend by study year *p *=* *.008

The prevalence of current asthma in 2003 was 8% (girls 8% vs. boys 8% *p *=* *.864) and in 2013, 11% (girls 12% vs. boys 11% *p *=* *.337). The proportion of adolescents who reported that their respiratory symptoms/asthma had an impact on daily life increased from 64% in 2003, to 70% in 2013 (test for trend, *p *=* *.008) (Figure 1). In 2013, the proportion reporting that asthma had an impact on daily life was higher among girls than boys (86% vs. 71%; test for trend *p *=* *.039). However, no differences according to sex were found in 2003 (data not shown).

**Figure 2:**
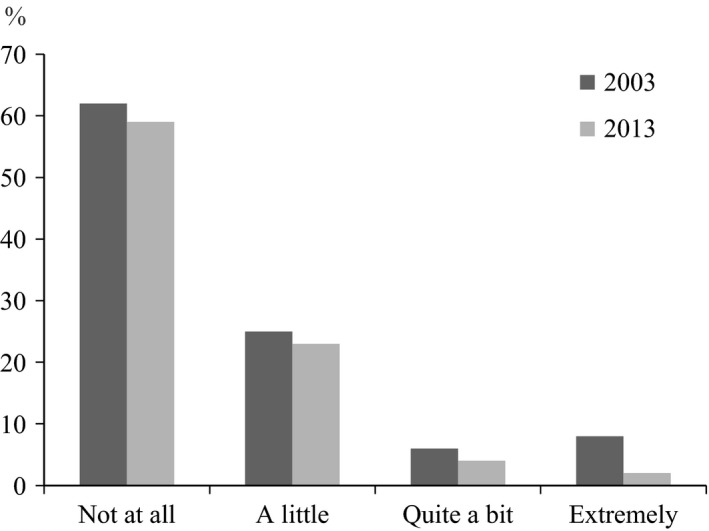
Proportion (%) of worsening of respiratory symptoms/asthma in school reported by adolescents with asthma (14–15 years old) in 2003 and 2013. Test for trend by study year *p *=* *.011

In both years, more than a quarter of the adolescents reported a worsening of respiratory symptoms/asthma at school (39% in 2003 vs. 29% in 2013; test for trend *p *=* *.011) (Figure 2). No significant differences by sex were found (data not shown). The proportion of school absenteeism due to respiratory symptoms/asthma was at a similar level in 2003 and 2013 (15% vs. 14%, *p *=* *.345), with no differences by sex (Table 1).

**Table 1:** Prevalence of self‐reported school absenteeism due to asthma and physical activity in relation to school among adolescents with asthma (14–15 years old) in 2003 and 2013

		All *n* (%)	*p* value^a^	Girls *n* (%)	Boys *n* (%)	*p* value^b^
Stayed home from school due to respiratory symptoms/asthma	2003 2013	40 (15.2) 32 (13.7)	.345	20 (15.6) 17 (14.2)	20 (14.8) 15 (13.2)	.855 .822
Do not having the same physical fitness as schoolmates	2003 2013	68 (25.8) 66 (28.0)	.920	35 (27.1) 44 (35.8)	33 (24.4) 22 (19.5)	.618 **.005**
Do not taking full part in physical education in school	2003 2013	37 (14.1) 27 (11.5)	.235	24 (18.6) 22 (18.2)	13 (9.8) 5 (4.4)	**.040** **.001**
Have respiratory symptoms/asthma associated with physical education, running or other sports^c^	2003	217 (83.1)		111 (86.7)	106 (79.7)	.130

The significant values are showing differences by sex in 2003 and 2013, respectively. This is clarified in the footnotes below.

a Difference by study year (2003 vs. 2013).

b Difference by sex in 2003 and 2013 respectively.

c Question only included in 2003.

### 4.2 Physical education at school

In 2003, a high proportion of the adolescents reported respiratory symptoms/asthma associated with physical education, running, or engaging in other sports (83%). The girls expressed discomfort to a greater extent than boys, however, not significantly (87% vs. 80%, *p *=* *.130) (Table 1; question only used in 2003). In both years, over a quarter of the adolescents reported that they did not have the same physical fitness as their schoolmates. Moreover, in 2013, a higher proportion of girls reported that they did not have the same physical fitness as their peers (36% girls vs. 20% boys, *p *=* *.005). In both years, the girls reported non‐participation in physical education to a higher extent than the boys (Table 1).

### 4.3 Triggers in the school environment

In 2003, 98 (39%) of the adolescents with current asthma reported a worsening of respiratory symptoms/asthma at school. Of these, 90 (92%) adolescents also completed the open follow‐up question, reporting asthma triggers in school. The content analysis resulted in seven categories describing the following triggers: poor air quality (sultry and dry air), a poorly cleaned environment (dust), allergens (peers with pets, mould, grass, food), strong fragrances (perfumes, smoke), building reconstructions, physical education and stress.

## 5 Discussion

A higher proportion of adolescents (14–15 years old) with current asthma reported that respiratory symptoms/asthma interfered in daily life in 2013 compared with 10 years earlier. Conversely, a decreased proportion reported a worsening of respiratory symptoms/asthma at school during the latter year. The proportion of adolescents reporting school absenteeism, not having the same physical fitness as the peers and not taking full part in physical education, did not change significantly over the 10 years. In 2013, girls seemed to be more affected by their respiratory symptoms/asthma with regard to daily life, physical fitness and physical education absenteeism than the boys. Asthma triggers at school were described to be poor air quality, a poorly cleaned environment, allergens, strong fragrances, building reconstructions, physical education and stress.

In the same cohort as described in the current paper Andersson, Bjerg, Forsberg, Lundbäck, and Rönmark (2010) compared the children with asthma in the two cohorts at the age of 8 years and found a decreased proportion of children with asthma reporting school absenteeism, 41–31%. In addition, absenteeism also decreased by age from 31% (8 year) (Andersson et al., 2010) to 14–15% in the same cohorts, but at the age of 14–15 as displayed in our study. This result supports previous findings that younger asthmatics are missing school more often than older ones (Bonilla et al., 2005). It is also well known that uncontrolled asthma is strongly associated with younger ages (Zahran, Bailey, Qin, & Moorman, 2014).

However, although school absenteeism due to asthma has decreased with increasing age, more than a quarter of the adolescents in our study, both in 2003 and 2013, still expressed that their asthma symptoms worsened at school. This is not surprising since measurements confirm contamination of cat and dog allergens in settled dust in the classrooms regardless of floor cleaning methods and ventilation (Kim et al., 2005; Perzanowski et al., 1999). In our study, when some of the adolescents reported different allergens as asthma triggers, several of the adolescents were bothered by furry animal allergens from classmates’ clothes. Other reported triggers that are difficult to avoid in the school environment included poor air quality, mould, a poorly cleaned environment and building reconstructions. As such, all staff members in a school have the responsibility to ensure that asthmatic children do not get worse in the school setting. They should therefore have knowledge about asthma, allergic diseases and factors that trigger symptoms (Borres et al., 2002).

In our study, we found that many of the adolescents in 2003 reported increased asthma symptoms related to physical education. Nonetheless, the majority indicated that they were still taking full part in physical activities in school. One explanation may be that asthmatic adolescents in Sweden are relatively well treated, which implies that they can participate in physical education with little or no restrictions. In line with earlier research (Chiang et al., 2006), asthmatic girls in our study participated to a lesser extent in physical education than asthmatic boys. However, the idea that asthma is the cause of the girls’ reduced participation should be interpreted cautiously. After all, studies about adolescents in school show that girls, in general, are less physically active than boys (Jago, Anderson, Baranowski, & Watson, 2005; Olds et al., 2009).

Earlier studies have shown that there are no differences in physical activity levels between children (7–16 years old) with or without asthma (Nystad et al., 2001). Despite that fact, our results revealed that in both 2003 and 2013, over 25% of the adolescents expressed that they did not have the same physical fitness as their peers and in 2013, there were also significant differences by sex with a higher proportion of girls. The girls also reported that their asthma interfered in daily life to a higher extent compared with the boys, suggesting that girls are more affected by the illness. This result is in line with earlier research, showing that health status is worse among asthmatic girls (Rydström et al., 2005; Sundell et al., 2011). The differences in sex may be an important factor to take into account when supporting adolescents with asthma during physical activities at school as well as supporting them in daily life.

### 5.1 Implications for healthcare professionals

Healthcare professionals meeting adolescents with asthma – for example, school nurses – have an important role to assist the adolescents to take command of their life with asthma (Englund, Hartman, & Segesten, 2006). Supporting asthmatic adolescents through education of self‐management has shown to improve feelings of self‐control, reduces absenteeism from school and number of days with restricted activity (Guevara, Wolf, Cyril, & Clark, 2003). In our study, the open question was based on the adolescents’ own experiences, allowing them to identify what in the school setting worsens their asthma. Self‐perceived answers are important and can help healthcare professionals to identify and manage asthma triggers in each school. The question: “If your asthma gets worse, what in the school setting do you think is causing the deterioration?” can, for example, be implemented in the adolescents’ individualized asthma management plans. Unfortunately, a survey of school nurses in the USA showed that 64% of the students with asthma were visiting the health office, but only 25% had a written management plan. Furthermore, more than half of the school nurses stated that they needed more information about asthma triggers (Kielb, Lin, & Hwang, 2007). A written individualized plan to manage asthma is an important tool that nurses in school can use to support the adolescents and give instructions related to the illness (Erickson, Splett, Mullett, Jensen, & Belseth, 2006). Therefore, our study highlights the need for using written management plans not only in healthcare settings, but also in school for asthmatic adolescents.

### 5.2 Strengths and limitations

A major strength of our study was the ability to compare two identical age cohorts, in the same geographical area, with an interval of 10 years and a high participation rate for both years. The high participation rate can be due to the fact that the adolescents answered the questionnaire in school during class. Another strength is the use of a well‐validated questionnaire, ISAAC and that the question of self‐reported asthma has been validated (Rönmark, Jonsson, Platts‐Mills, & Lundbäck, 1999). One limitation is that two of the questions, were only used in 2003, making it impossible to analysis changes over time. However, regarding the open question about asthma triggers in school, research shows that improvement in the school environment during the said years has been minor. In fact, a review from 2013 about indoor pollution in school showed that schools in general still have severe indoor air problems due to poor building constructions, poor cleaning, poor ventilation and high levels of allergens and moulds (Annesi‐Maesano, Baiz, Banerjee, Rudnai, & Rive, 2013). Nevertheless, a recommendation for further research is conducting qualitative interview studies on asthmatic adolescents’ experiences about triggers in the school environment.

## 6 Conclusion

In summary, this study showed that still in 2013, asthma affects adolescents’ daily lives in school and both school attendance and physical education are negatively affected by the disease and its symptoms. However, the open question showed that adolescents can identify asthma triggers in their school settings, which is important information to consider at each school. This study indicates that healthcare professionals may improve the daily lives of asthmatic adolescents in school with support, education and the use of an individualized asthma management plan to identify asthma triggers in the school environment.

## Author contribution

Linnéa Hedman was responsible for the study design. Caroline Stridsman, Elisabeth Dahlberg and Karin Zandrén performed the data analysis. All authors were responsible for drafting the manuscript and gave final approval of the manuscript to be published.
